# Adipose tissue is the first colonization site of *Leptospira interrogans* in subcutaneously infected hamsters

**DOI:** 10.1371/journal.pone.0172973

**Published:** 2017-02-28

**Authors:** Ryo Ozuru, Mitsumasa Saito, Takaaki Kanemaru, Satoshi Miyahara, Sharon Y. A. M. Villanueva, Gerald L. Murray, Ben Adler, Jun Fujii, Shin-ichi Yoshida

**Affiliations:** 1 Department of Bacteriology, Faculty of Medical Sciences, Kyushu University, Fukuoka, Japan; 2 Division of Bacteriology, Department of Microbiology and Immunology, Faculty of Medicine, Tottori University, Tottori, Japan; 3 Department of Microbiology, University of Occupational and Environmental Health, Fukuoka, Japan; 4 Morphology Core Unit, Kyushu University Hospital, Fukuoka, Japan; 5 Department of Microbiology, Monash University, Melbourne, Australia; University of Toledo College of Medicine and Life Sciences, UNITED STATES

## Abstract

Leptospirosis is one of the most widespread zoonoses in the world, and its most severe form in humans, “Weil’s disease,” may lead to jaundice, hemorrhage, renal failure, pulmonary hemorrhage syndrome, and sometimes,fatal multiple organ failure. Although the mechanisms underlying jaundice in leptospirosis have been gradually unraveled, the pathophysiology and distribution of leptospires during the early stage of infection are not well understood. Therefore, we investigated the hamster leptospirosis model, which is the accepted animal model of human Weil’s disease, by using an *in vivo* imaging system to observe the whole bodies of animals infected with *Leptospira interrogans* and to identify the colonization and growth sites of the leptospires during the early phase of infection. Hamsters, infected subcutaneously with 10^4^ bioluminescent leptospires, were analyzed by *in vivo* imaging, organ culture, and microscopy. The results showed that the luminescence from the leptospires spread through each hamster’s body sequentially. The luminescence was first detected at the injection site only, and finally spread to the central abdomen, in the liver area. Additionally, the luminescence observed in the adipose tissue was the earliest detectable compared with the other organs, indicating that the leptospires colonized the adipose tissue at the early stage of leptospirosis. Adipose tissue cultures of the leptospires became positive earlier than the blood cultures. Microscopic analysis revealed that the leptospires colonized the inner walls of the blood vessels in the adipose tissue. In conclusion, this is the first study to report that adipose tissue is an important colonization site for leptospires, as demonstrated by microscopy and culture analyses of adipose tissue in the hamster model of Weil’s disease.

## Introduction

Leptospirosis, which is mainly caused by the pathogenic spirochete *Leptospira interrogans*, is one of the most widespread and potentially fatal zoonoses in the world [1]. Many wild and domestic animals can serve as reservoir hosts for leptospires and the brown rat (*Rattus norvegicus*) is the most important source of human infections [2]. Rat urine containing leptospires contaminates environmental soil and water, and leptospires infect humans and livestock when they are exposed to a contaminated environment [3]. The early stage of leptospirosis is similar to that of other febrile illnesses, but the most severe clinical manifestations of leptospirosis are jaundice, renal failure and hemorrhage (Weil’s disease) and sometimes pulmonary hemorrhage syndrome [4–6]. Because *L*. *interrogans* has more than 250 serovars [2], effective universal vaccination is not yet available.

Leptospires infect humans percutaneously or across mucous membranes by penetrating abraded skin and/or mucous membrane [7], move immediately into the bloodstream and cause bacteremia [2]. After leptospires reach the liver, which is one of the major target organs for jaundice [8, 9], they destroy the hepatocellular junction, disrupt the bile canaliculi, and then cause jaundice [10]. Although the mechanisms underlying jaundice in leptospirosis have been gradually unraveled, distribution of leptospires and the pathogenesis during the early stage of infection are poorly understood. This knowledge gap must be solved so that early measures for arresting the progression of infection into Weil’s disease can be implemented. Until now, *Leptospira* culture from infected organs or quantitative polymerase chain reaction analysis of leptospiral DNA have been performed to detect *Leptospira* in the target organs of experimentally infected animals [11, 12]. It is still not known, however, whether leptospires colonize non-target organs. In this study, therefore, we chose an *in vivo* imaging system (IVIS) to observe the distribution of leptospires in the whole bodies of infected animals.

Nowadays, IVIS is frequently used for following stem cell and bacterial dynamics in live animals [13]. To analyze microbial infections in animals with IVIS, luminescent or fluorescently labeled microbes are used [14]. In the 2000s, genetic tools for *Leptospira* spp. were developed [15], and our group constructed a luminescent *Leptospira* line (strain M1307) [16]. Transposon Tn*SC189*, which was modified to incorporate the *luxCDABE* cassette from *Photorhabdus luminescens* [17] and the constitutive *flgB* promoter from *Borrelia burgdorferi* [18, 19], was used to construct luminescent *L*. *interrogans* [20]. After this, two other teams also succeeded in constructing genetically modified *Leptospira* strains expressing either fluorescent GFP or mRFP proteins, or firefly luciferase [21, 22]. To analyze leptospirosis, some *in vivo* imaging studies were performed using zebrafish and mouse models of infection [22, 23]. These studies revealed the quick uptake of leptospires by phagocytic cells in transparent zebrafish embryos [23]. It was also established that the novel bioluminescent *L*. *interrogans* mouse infection model provided a powerful way to study both acute and chronic leptospirosis and, notably, revealed that leptospires could escape antibody attack by residing in the kidneys [22].

Golden Syrian hamsters are highly susceptible to leptospiral infection and are considered to be good animal models for leptospirosis [24, 25]. The manifestations and severity of infection in hamsters are similar to Weil’s disease in humans; thus, these animals are preferred in most leptospirosis animal studies [26]. However, as far as we know, IVIS analysis has not been performed in the hamster model of leptospirosis. In this study, we analyzed M1307-infected hamsters using IVIS, immunohistochemistry, and electron microscopy.

## Materials and methods

### Ethics statement

Animal experiments were reviewed and approved by the Ethics Committee on Animal Experiments at the Faculty of Medical Sciences, Kyushu University (Permit Number: A26-057). The experiments were carried out under the conditions indicated in the Regulations for Animal Experiments of Kyushu University and law 105 and notification 6 of the Government of Japan.

### Bacterial culture and infection experiments

In this study, we used *L*. *interrogans* serovar Manilae strain M1307, derived from the parent strain L495 by insertion of the *luxCDABE* cassette from plasmid pSB406 obtained from Dr. S. Swift, University of Nottingham, UK [16, 17]. The promoter was the *flgB* constitutive promoter from *Borrelia burgdorferi* [18, 19]. The parent strain L495 was isolated from rat kidney in the Philippines and was obtained from Dr. N. Koizumi, National Institute of Infectious Diseases, Tokyo, Japan. M1307 was grown in Korthof’s medium [27] with kanamycin (25 μg/ml) at 30°C without shaking. Leptospires were counted using a Thoma cell counting chamber (Sunlead Glass Corp., Saitama, Japan).

For the infection experiments, 8-week-old female Golden Syrian hamsters were purchased from Japan SLC Inc. (Shizuoka, Japan). M1307 (10^4^) in 100 μl of Korthof’s medium or 100 μl of Korthof’s medium without leptospires was injected subcutaneously into the right inguinal region of hamsters from the experimental (n = 8) or uninfected control (n = 2) groups, respectively. Hamsters were observed for 14 days, and moribund animals were euthanized by cervical dislocation under anesthesia in accordance with the animal ethics guidelines. Excised organs from the infected hamsters were homogenized with disposable syringes and then cultured in Korthof’s medium containing a combination of five antimicrobials (sulfamethoxazole, trimethoprim, amphotericin B, fosfomycin, and 5-fluorouracil) [28] at 30°C. *Leptospira* growth in these cultures was monitored by dark-field microscopy every day for 7 days.

### Bioluminescent imaging

Bioluminescent imaging by IVIS was conducted as described previously [22]. For whole body imaging, a total of 10 hamsters were infected with 10^4^ *L*. *interrogans* M1307 or injected with Korthof’s medium only. The infected hamsters were anesthetized with isoflurane by inhalation and placed in an acrylic plastic box. Bioluminescent images of the hamsters were then acquired using an IVIS (Spectrum, PerkinElmer, Inc., Waltham, MA; using settings of exposure time 5 sec, medium binning, F/Stop = 1). Data acquisition and analysis were performed using Living Image 4.4 software (PerkinElmer, Inc.). Quantification was performed using a region of interest (ROI) defined manually (abdominal center or injection site), and the results were expressed as photons (P) per second (s). All the acquired images and defined ROIs are shown in S1 Fig.

Skin incision, laparotomy and subsequent *ex vivo* analyses were performed to obtain more precise bioluminescent images. A total of 21 hamsters were infected with 10^4^ *L*. *interrogans* M1307. The hamsters (n = 3) were imaged with the IVIS (all the acquired images are shown in S2 Fig) and euthanized by cervical dislocation under anesthesia each day. After that, skin incision and laparotomy were performed and the bioluminescent images were obtained. Blood was collected through cardiac puncture and one drop from each hamster was used for imaging. Livers and kidneys were removed, and transverse and longitudinal images were obtained. All the acquired images of blood, livers and kidneys are shown in S3 Fig. To avoid the loss of luminescence activity, one hamster was imaged at a time. For all the analyses, we used settings of exposure time 5 sec, medium binning, F/Stop = 1.

### Microscopy

Microscopy was performed as described previously [10]. To observe the skin and subcutaneous tissues, infected hamsters were euthanized at day 6 post-infection. Whole bodies were perfused from the left ventricle with 0.5% heparin in phosphate-buffered saline (PBS).

#### Immunofluorescent microscopy

Perfused whole bodies were then fixed with a mixture of 4% paraformaldehyde and 0.1% glutaraldehyde in PBS. After excision, the skin with adipose tissue was cut (5×5 mm) and washed with PBS. The cut skin was immersed in 10–20% sucrose overnight, placed in isopentane, and frozen on a metal plate chilled with liquid nitrogen. Each piece of skin was then sliced to a thickness of 60 μm using a cryotome (Laika, Germany) and then mounted on a microscopic slide. The slices were blocked with 3% bovine serum albumin in PBS (blocking buffer), washed with PBS, and incubated with a rabbit anti-serovar Manilae antiserum (1:200) at 4°C overnight [26]. After washing with PBS, the slices were incubated with a goat anti-rabbit Cy5 antibody (1:500; Molecular Probes Inc., Eugene, OR, USA) for 1 h at room temperature to stain the bacteria. Stained slices were washed with PBS, stained with 2 μg/ml 4′,6-diamidino-2-phenylindole (DAPI), and then observed with a light-field microscope and a Confocal Microscope A1 (Nikon Corporation, Tokyo, Japan).

#### Transmission electron microscopy

After perfusion and fixation of the whole hamster bodies with a mixture of 2.5% paraformaldehyde and 2% glutaraldehyde in PBS, the adipose tissues were cut into 2×2×10 mm pieces and kept in 2% glutaraldehyde at 4°C. The pieces were fixed with 4% osmium tetroxide for 45 min and then stained with uranyl acetate for 1 h, after which they were embedded in Epon and sliced with an ultramicrotome. The samples were observed with an HT7700 transmission electron microscope (Hitachi, Tokyo, Japan).

### Statistical analysis

Statistical analysis was performed using JMP Pro 11.0.0 (SAS Institute Inc.). The unpaired *t* test (two-tailed P values) was used to compare two groups at the same time point. Values are expressed as mean ± standard error of the mean (SEM). A *p* value <0.05 was considered significant. *p* values: *p<0.05

## Results

### Progression of M1307 bioluminescence in infected hamsters *in vivo*

**Fig 1:**
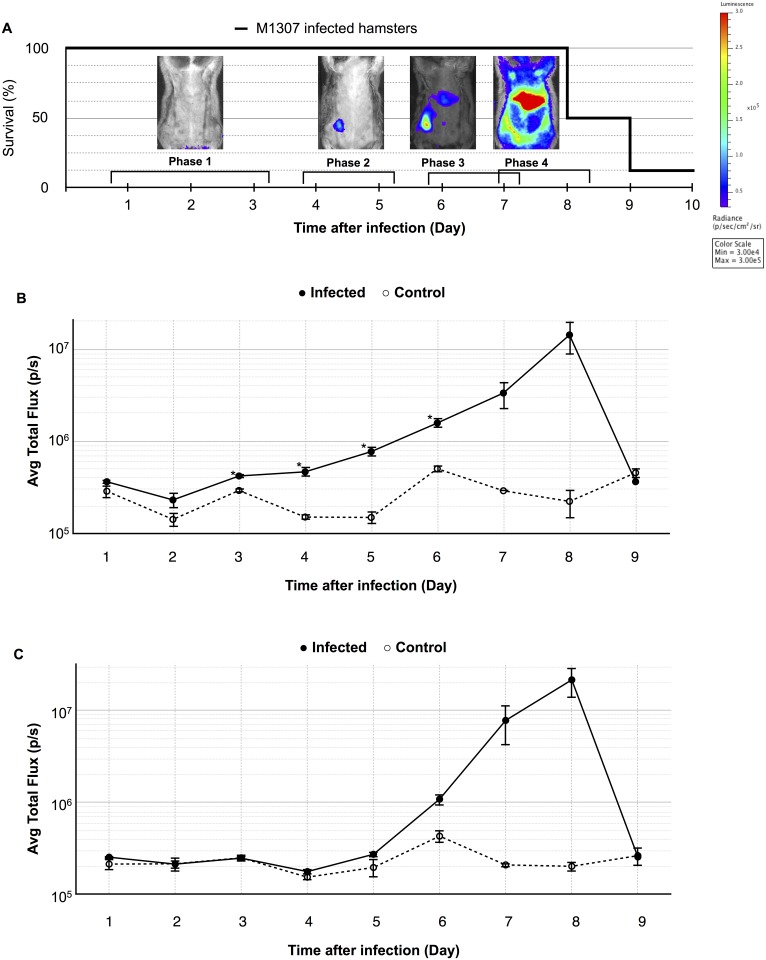
Bioluminescence dissemination of *Leptospira* in hamsters. (A) The survival rate of Golden Syrian hamsters (n = 8) infected subcutaneously with 10^4^ *L*. *interrogans* strain M1307 into the right inguinal region, and representative ventral view photographic images tracking the hamster infections on different days post-infection. Images depict photographs overlaid with color representations of luminescence intensity, measured in photons/second/cm^2^/sr as indicated on the scales, where red is the most intense (3×10^5^) and purple is the least intense (3×10^4^). (B,C) Average luminescence intensities in each ROI of injection site (B) and abdominal center (C) at different days post-infection. Data are expressed as the means ± SEM of total flux in photons/second in each ROI in eight infected hamsters (●) and two uninfected controls (◦). *p* values (*p<0.05), between groups.

Golden Syrian hamsters were infected subcutaneously with 1 × 10^4^ *L*. *interrogans* luminescent strain M1307. Seven of the eight infected hamsters (87.5%) required euthanasia by day 9 post-infection (Fig 1A). Based on our previous findings, we confirmed that the pathogenicity of strain M1307 was almost the same as that of the wild type strain (serovar Manilae) [16, 26]. Each day, luminescence from the hamsters was measured by IVIS; representative images are shown in Fig 1A. Until day 3 post-infection, there was no luminescence detected. On days 4 and 5, luminescence was detectable in the injection area only. On day 6, luminescence at the center of the abdominal area was observed. Eventually, on day 7 and 8, luminescence could be detected in the whole body of 4 of 8 hamsters (S1 Fig). The average total flux of injection site or the center of abdomen of the eight infected hamsters and two control hamsters were plotted and are shown in Fig 1B and 1C.

Based on these results, the four phases in the progression of leptospiral infection were defined as follows: Phase 1, no luminescence was detected (days 1 to 3); Phase 2, luminescence was detected at the injection site only (days 4 to 5); Phase 3, luminescence was detected in the injection area and the center of the abdomen (day 6 to 7); and Phase 4, luminescence was detected in the greater part of the body (day 7 to 8).

### Spread of luminescent *L*. *interrogans* in hamster organs

**Fig 2:**
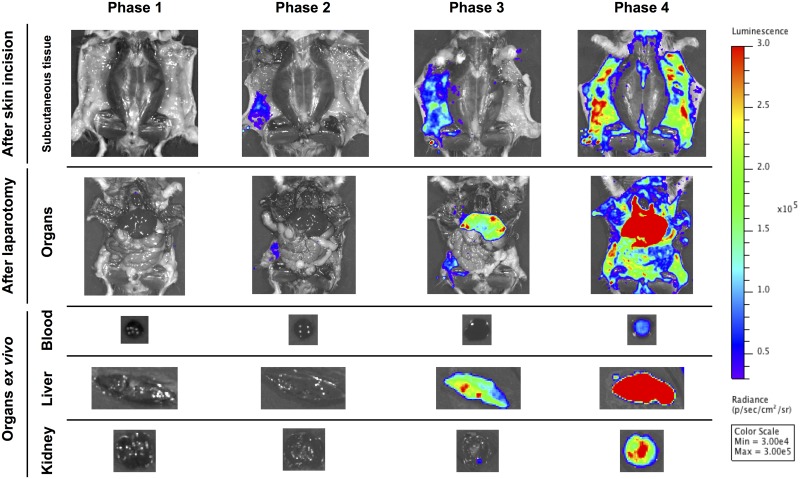
Bioluminescence changes in hamster organs. Representative bioluminescence images (ventral view) from M1307-infected hamsters at each phase. Images represent subcutaneous tissues after skin incision and organs after laparotomy, as well as *ex vivo* organs (blood plus liver and kidney cross sections). The scale is the same as in Fig 1.

To observe directly the organs of the M1307-infected hamsters by IVIS, we made skin incisions and performed laparotomies and *ex vivo* examinations to identify which organs were colonized by *Leptospira* cells. Therefore, we acquired five types of images during each phase: i) subcutaneous tissue after skin incision; ii) organs after laparotomy; and iii) to v) organs *ex vivo* (blood, cross sections of the liver and kidneys) (Fig 2). There was no luminescence during phase 1. Then, the luminescence became detectable only in the subcutaneous tissue during phase 2. In phase 3, luminescence in the liver was detectable and was stronger than that in the subcutaneous tissues. Finally, the luminescence was detected in the greater part of the body (including the blood and kidneys) during phase 4.

### Leptospiral culture in adipose tissue and other tissues

After careful macroscopic observation of the distribution of luminescence in the subcutaneous tissue, we identified it as subcutaneous adipose tissue. So far, there are no reports on adipose tissue cultures from animals experimentally infected with *Leptospira*. We therefore cultured adipose tissue from the infected animals as well as from their blood, livers, and kidneys (Table 1). It was found that some blood, liver and kidney cultures became positive during days 1, 3, and 4, respectively, and all cultures of each organ became positive at days 4, 4, and 7, respectively. In contrast, the adipose tissue cultures became *Leptospira*-positive during the first day post-infection and all of them became positive during day 2, which preceded the other organ cultures. These data suggest that during the early stage of leptospirosis, *Leptospira* colonize and grow in hamster adipose tissues.

**Table 1:** Number of culture-positive blood or organs collected from three hamsters infected with M1307.

Organs	Time after infection (Day)						
	1	2	3	4	5	6	7
**Adipose tissue**	1	3	3	3	3	3	3
**Blood**	1	1	2	3	3	3	3
**Liver**	0	0	1	3	3	3	3
**Kidney**	0	0	0	1	1	1	3

### *Leptospira* are observed in adipose tissue blood vessels

**Fig 3:**
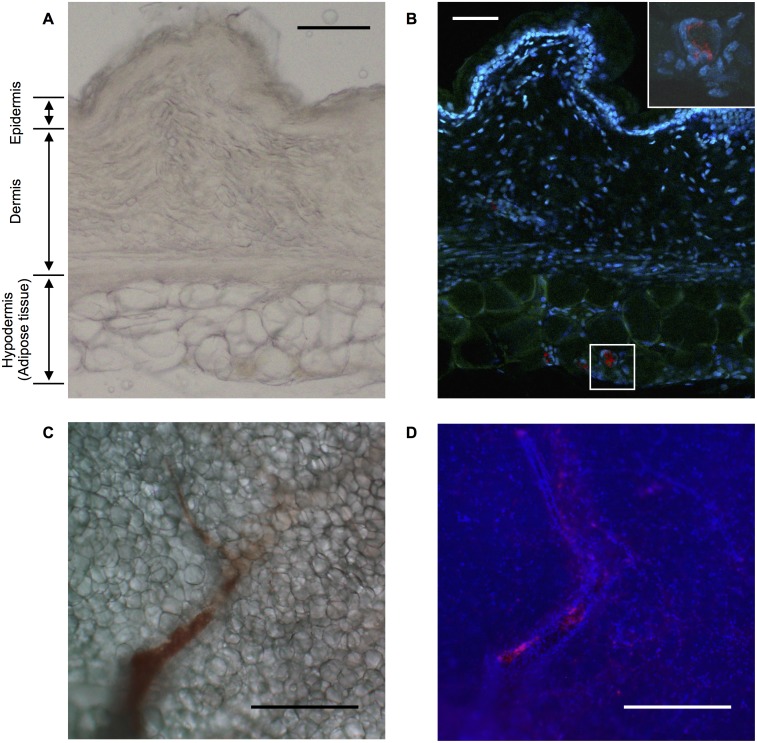
*Leptospira* distribution in skin and subcutaneous tissue. Representative light field (A, C) and fluorescence images (B, D) of the skin and subcutaneous tissue (A, B) or adipose tissue (C, D) around the injection sites of M1307 collected from infected hamsters at phase 4. Fluorescence images (B, D) showing cell nuclei stained with DAPI (blue), autofluorescence of the skin and subcutaneous tissue (green, not shown in panel D), and leptospires stained with rabbit polyclonal antiserum and Cy5-conjugated anti-rabbit monoclonal antibody (red). The framed area in (B) is enlarged at the upper right. Scale bars: 100 μm (A, B), 500 μm (C, D).

**Fig 4:**
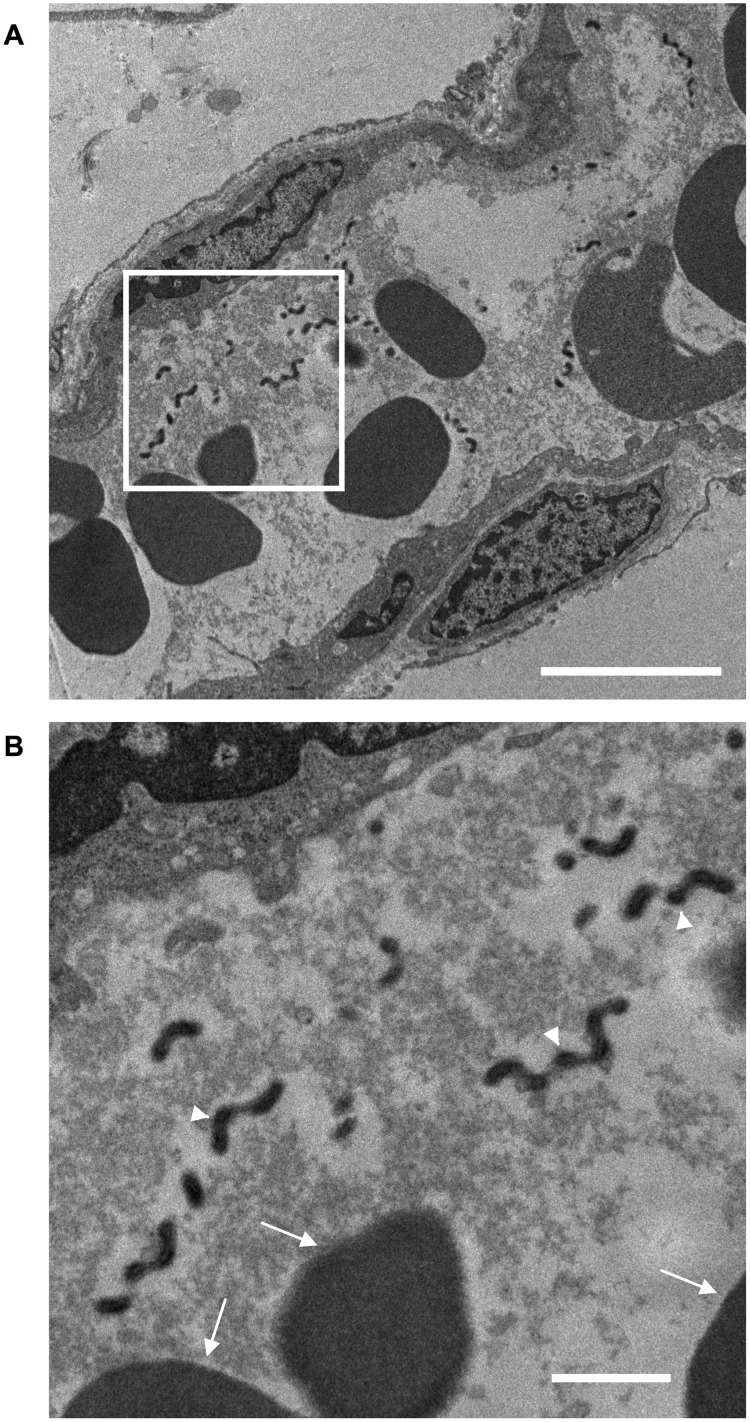
Transmission electron microscopy of adipose tissue blood vessels. Representative transmission electron microscope images of subcutaneous adipose tissue blood vessels around the injection sites of *Leptospira*-infected hamsters at phase 4. The framed area in (A) is enlarged in (B). The scale bars represent 5 μm (A) and 1 μm (B). The arrowheads point to *Leptospira* and the arrows show the red blood cells.

To confirm the presence of *Leptospira* cells in hamster adipose tissue, immunofluorescent microscopy of the skin was performed. Light-field images of the skin indicated that the epidermis, dermis and hypodermis including subcutaneous adipose tissue (Fig 3A). In the immunofluorescent images, labeled *Leptospira* cells were observed in the hypodermis including subcutaneous adipose tissues, but not in the epidermis or dermis (Fig 3B). Interestingly, *Leptospira* cells were observed nearby only in the area of dense nuclei but not adipocytes. To examine the location(s) of the leptospires in the adipose tissue, confocal laser scanning microscopy was performed. These images show that *Leptospira* colonized along the inner walls of the blood vessels in the adipose tissues (Fig 3C and 3D). To confirm our observations, we studied the blood vessels by transmission electron microscopy. *Leptospira* cells were found in the blood vessels (Fig 4) and coexisted always with the red blood cells though perfused with heparin in PBS. Interestingly, *Leptospira* was also always surrounded by a hazy fibrin-like structure. The bacterium was not observed within the adipocytes themselves (data not shown).

## Discussion

We performed this study to determine where in the body leptospires colonize and grow at the early phase of an infection. Although the clinical manifestations of leptospirosis are relatively mild, they may eventually worsen and become lethal if appropriate therapy is not initiated. It is already known that when pathogenic leptospires infect percutaneously, they immediately move into the bloodstream and persist there during the leptospiremic phase of the illness [2, 25]. Our study is the first report suggesting that leptospires initially colonize the blood vessels of adipose tissues in the hamster model of Weil’s disease, as based on the results of IVIS, microscopy, and culture.

IVIS is a useful tool for long-term observation of the whole bodies of living animals infected with pathogens [14]. In our observations using IVIS, leptospiral infection in the hamsters progressed stepwise. After sufficient growth in the adipose tissue at the early step of infection, the leptospires then reached the liver, the major target organ, via the bloodstream (Fig 2). This was shown by the finding wherein the liver was the last organ to have the strongest luminescence. Finally, the greater part of the body of each hamster was invaded by leptospires (Fig 1 Phase 4 and Fig 2 Phase 4).

We found that the luminescence faded immediately after the hamsters were euthanized though the luminescence could be detected immediately before death. Animals still alive while undergoing IVIS will of course take in oxygen with the anesthetic gas. In the experiment for Fig 2, the hamsters were examined post-euthanasia, when subsequent oxygen usage by the liver should have decreased. The luminescence in the liver during phases 3 and 4 was not detected when livers were covered with the peritoneum, but was detected after laparotomy. We surmise that the livers remained anaerobic before laparotomy, but they became aerobic when exposed to oxygen after laparotomy. Because one of the substrates for the bioluminescent reaction of *luxCDABE* is molecular oxygen [20], this would explain why the luminescence in the liver was detected only after laparotomy (Fig 2).

In this study, we combined IVIS (Figs 1 and 2) and tissue culture (Table 1) analyses to obtain valid evidence of leptospiral colonization in the infected animals. Although leptospires were culturable in blood from the beginning of the IVIS experiment, the luminescence level in the blood was very low throughout the course of infection (Fig 2, Table 1). This indicates that most of the leptospires in the host were tightly colonizing in the blood vessels of the adipose tissue, and only a few leptospires were free in the bloodstream.

Our findings are consistent with the fact that the essential carbon source for leptospiral growth is long-chain fatty acids [29]. Some studies have reported on the relationships between leptospires and fatty acids, mainly as growth factors [30,31] or chemoattractants [32]. *L*. *interrogans*, a pathogenic species, shows chemotactic behavior towards 100 mM palmitic acid 16 times stronger than that recorded for *L*. *biflexa*, a nonpathogenic species. We also tested whether leptospires display chemotaxis towards oleic acid using a microscopic agar-drop assay [33], but no chemotactic behavior was detected (data not shown), probably because oleic acid is toxic to leptospires [34].

In the animal experiments we have described, only one infected hamster survived the infection (Fig 1). In this hamster, the luminescence at the injection site was detected once but it was not detected at the central abdomen throughout the experiment (S1 Fig, No. 8). This result suggests that the leptospires were eliminated from the infected area and did not reach the liver to cause fatal pathology in this survivor, and may be the reason why there were slight differences between infected group and control group at the injection area at day 7 and 8 (*p* = 0.0502 and 0.1052 respectively, Fig 1B) and on abdominal center at day 6, 7 and 8 (*p* = 0.0760, 0.0896 and 0.1052 respectively, Fig 1C).

Intriguingly, in microscopic observations of the adipose tissue blood vessels in the infected hamsters, leptospires were detected only in a hazy structure with the red blood cells (Fig 4). This hazy structure is suggestive of the first step in blood clotting, and fibrin might be a component of the haze-like structure. This observation is based only on the hamsters at phase 4. Therefore, the time-dependency of when the clot was formed around the leptospiral organisms in the blood vessels is unknown.

Besides adding to conventional knowledge about the transition of *Leptospira* cells into the bloodstream immediately after infection, this study shows a component of the pathological mechanisms of leptospiral infection during the early step whereby, in the hamster infection model, leptospires colonize the blood vessel inner walls of the adipose tissue. This is the first demonstration of the significant involvement played by the adipose tissues during the early stage of leptospiral infection.

## Supporting information

S1 Fig All the acquired images about luminescence from M1307 infected hamsters with IVIS (for analyzing survival rate and changes of luminescence). Circles represent ROIs in the injection site (blue) or the abdominal center (red).
(TIF)

S2 Fig All the acquired images about luminescence from M1307 infected hamsters with IVIS (before skin incision, laparotomy and *ex vivo* analyses). The 3 hamsters were performed skin incision, laparotomy and subsequent *ex vivo* analyses each day.
(TIF)

S3 Fig All the acquired luminescence images from *ex vivo* organs of M1307 infected hamsters with IVIS. (TIF)
