# Supplementary figures and images for: Adipose tissue is the first colonization site of Leptospira interrogans in subcutaneously infected hamsters
Source: PLoS One. 2017 Feb 28;12(2):e0172973. doi: 10.1371/journal.pone.0172973 (PMC5330501; doi:10.1371/journal.pone.0172973)

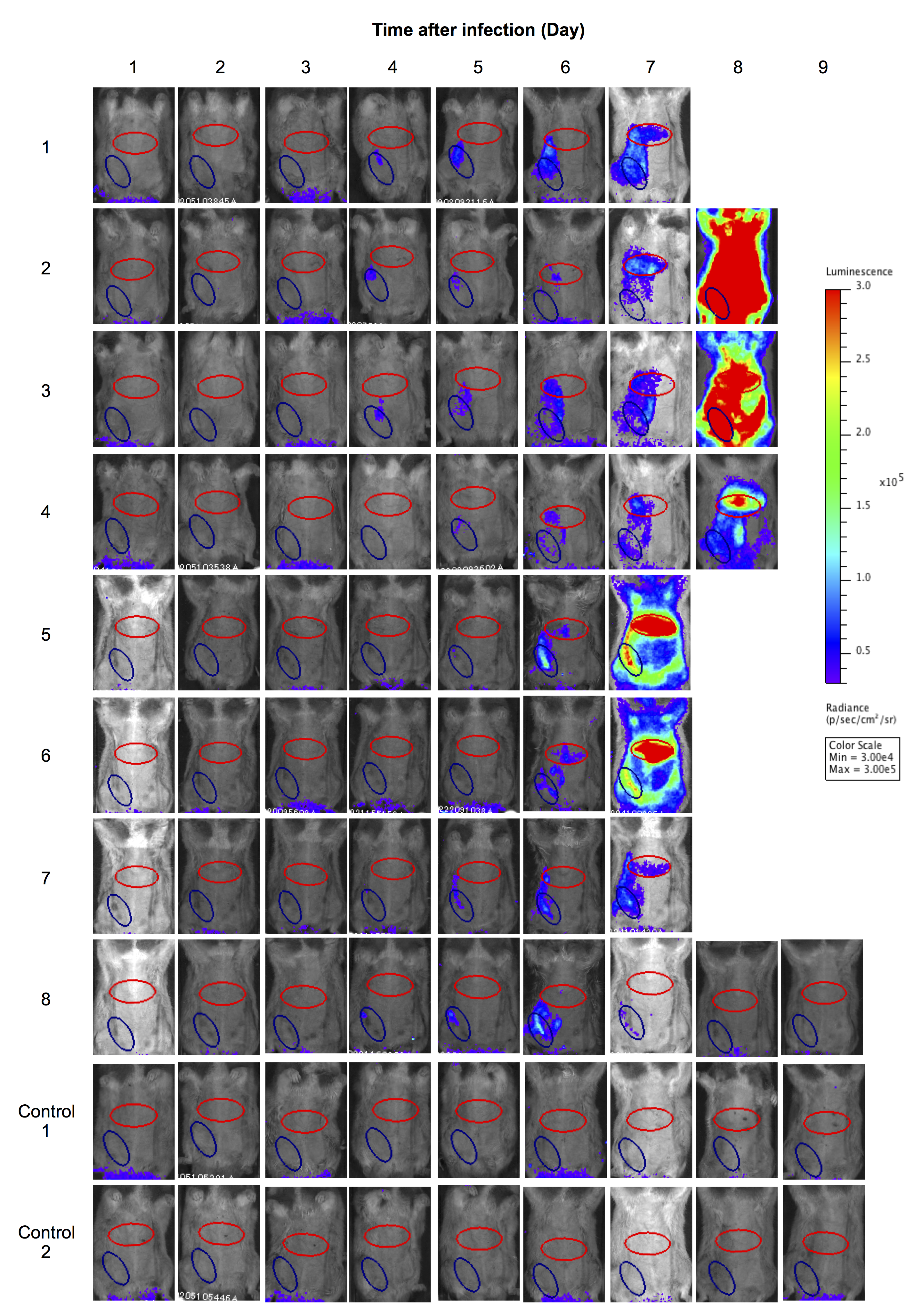

Supplement: S1 Fig — Circles represent ROIs in the injection site (blue) or the abdominal center (red). (TIF) [file pone.0172973.s001.tif]

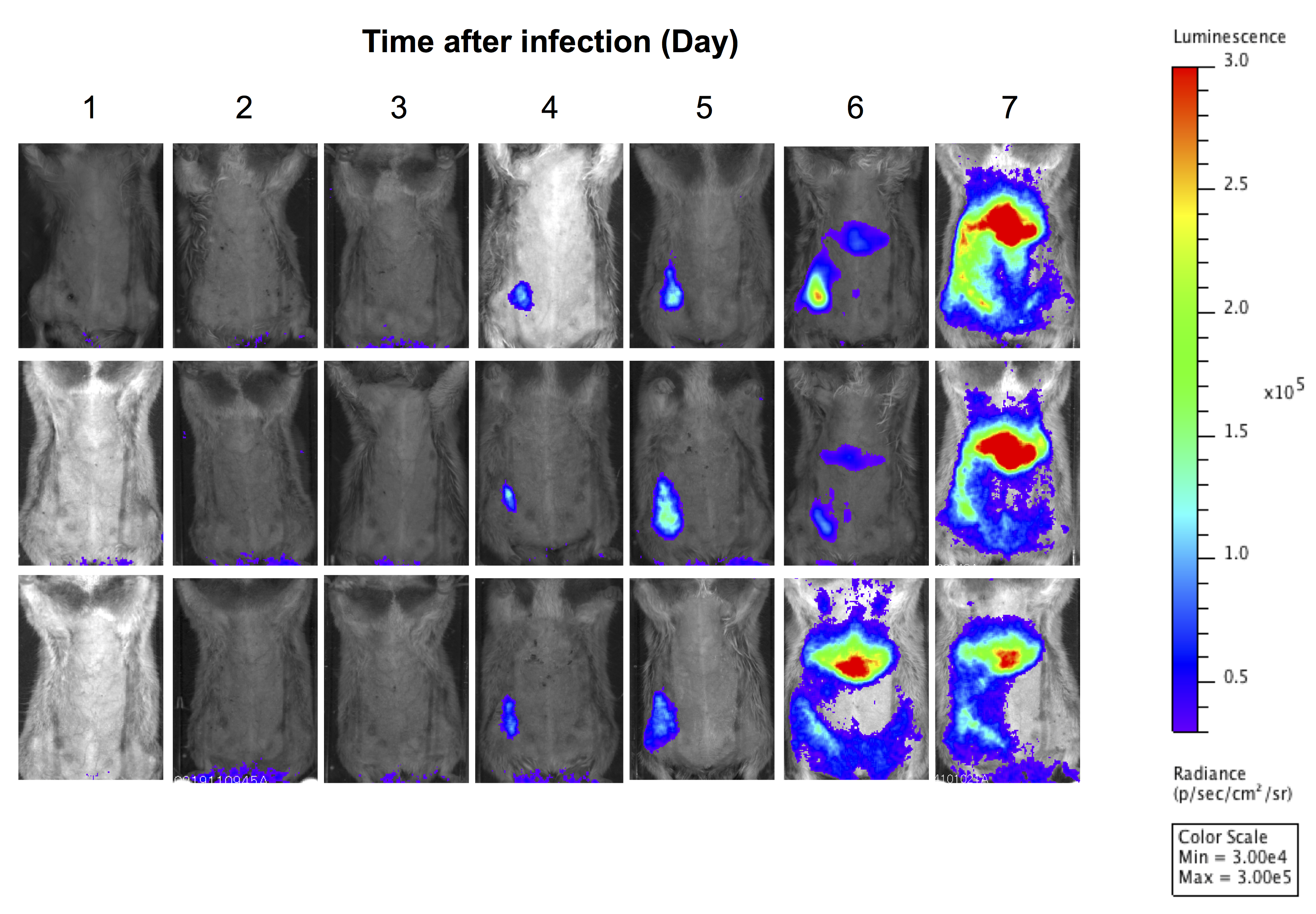

Supplement: S2 Fig — The 3 hamsters were performed skin incision, laparotomy and subsequent ex vivo analyses each day. (TIF) [file pone.0172973.s002.tif]

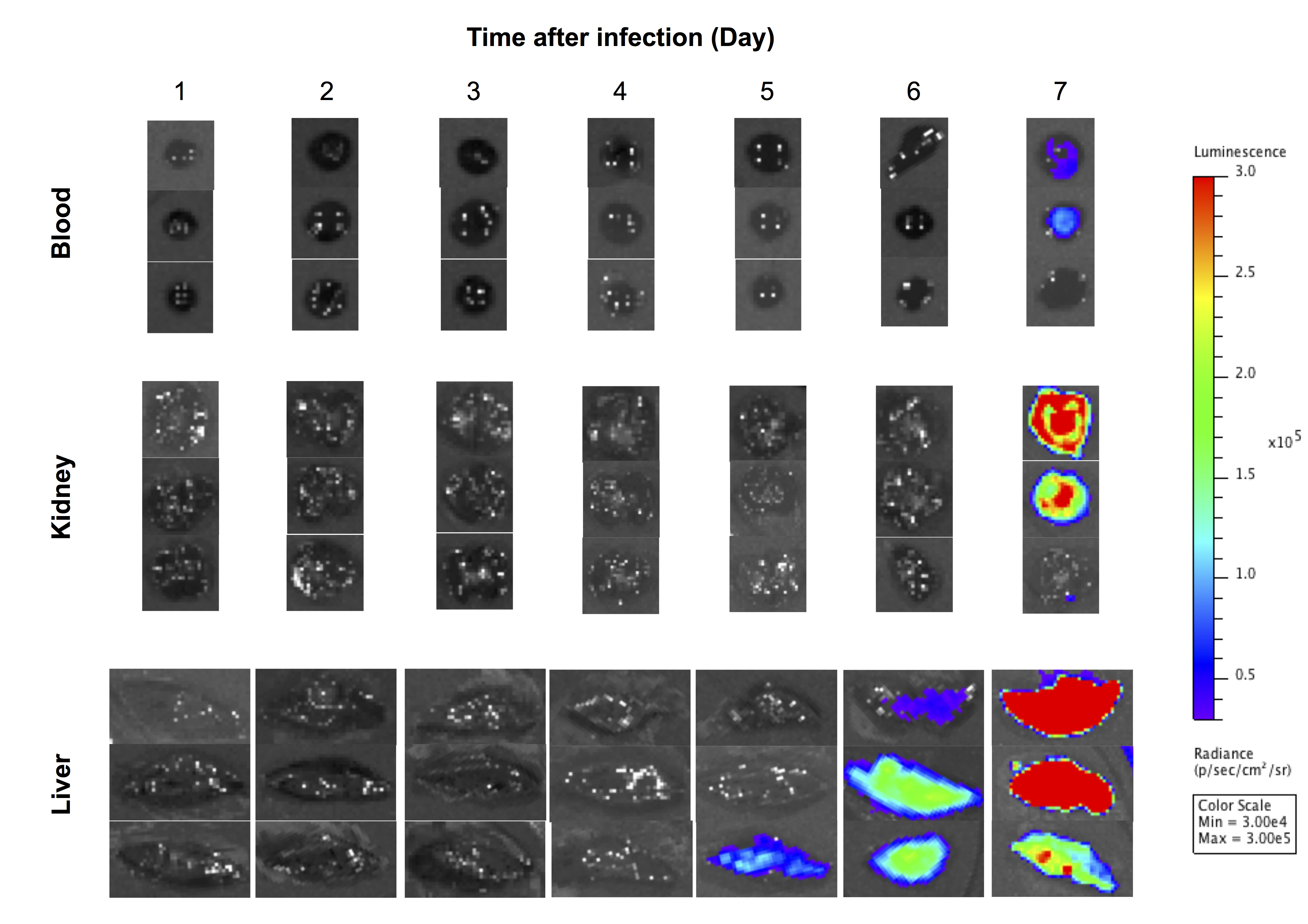

Supplement: S3 Fig — (TIF) [file pone.0172973.s003.tif]
